# National targets, process transformation and local consequences in an NHS emergency department (ED): a qualitative study

**DOI:** 10.1186/1471-227X-14-12

**Published:** 2014-06-13

**Authors:** Paraskevas Vezyridis, Stephen Timmons

**Affiliations:** 1 School of Health Sciences, Frederick University Cyprus, 7 Y. Frederickou Str, Nicosia 1036, Cyprus; 2 Faculty of Medicine & Health Sciences, University of Nottingham, Queen's Medical Centre, Nottingham NG7 2UH, UK

**Keywords:** Emergency department, Wait target, Built environment, Information technology, Professional values

## Abstract

**Background:**

In the attempt to reduce waiting times in emergency departments, various national health services have used benchmarking and the optimisation of patient flows. The aim of this study was to examine staff attitudes and experience of providing emergency care following the introduction of a 4 hour wait target, focusing on clinical, organisational and spatial issues.

**Methods:**

A qualitative research design was used and semi-structured interviews were conducted with 28 clinical, managerial and administrative staff members working in an inner-city emergency department. A thematic analysis method was employed and NVivo 8 qualitative data analysis software was used to code and manage the emerging themes.

**Results:**

The wait target came to regulate the individual and collective timescales of healthcare work. It has compartmentalised the previous unitary network of emergency department clinicians and their workspace. It has also speeded up clinical performance and patient throughput. It has disturbed professional hierarchies and facilitated the development of new professional roles. A new clinical information system complemented these reconfigurations by supporting advanced patient tracking, better awareness of time, and continuous, real-time management of emergency department staff. The interviewees had concerns that this target-oriented way of working forces them to have a less personal relationship with their patients.

**Conclusions:**

The imposition of a wait-target in response to a perceived “crisis” of patients’ dissatisfaction led to the development of a new and sophisticated way of working in the emergency department, but with deep and unintended consequences. We show that there is a dynamic interrelation of the social and the technical in the complex environment of the ED. While the 4 hour wait target raised the profile of the emergency department in the hospital, the added pressure on clinicians has caused some concerns over the future of their relationships with their patients and colleagues. To improve the sustainability of such sudden changes in policy direction, it is important to address clinicians’ experience and satisfaction.

## Background

Prolonged patient wait times in NHS emergency departments (EDs), here defined as the number of minutes between the time the patient arrives at the ED and the time the patient is admitted, transferred or discharged from the ED (length of stay), have traditionally been a major cause of patient dissatisfaction [1-5], particularly as the demand for emergency care is rising and acuity is becoming increasingly complex [6]. They are also a cause of patients leaving the ED before being seen by a clinician [7-9], adverse events [10], restricted access to emergency care [11] and increased mortality rates [12].

To address these chronic problems in EDs, wait targets have been applied as a means to monitor, assess and, therefore, improve the overall experience and quality of care. The focus on targets has triggered controversy about their effectiveness [13-20]. Findings from a recent systematic review [21], suggest that the 4 hour ED wait target in the English NHS has failed to consistently improve clinical outcomes and cautions countries which have embarked upon similar schemes [22,23] to learn these lessons. Certainly, these targets can speed up the patients’ journey through the ED [24,25], particularly as they concentrate organisational and clinical efforts in meeting them [26-30]. However, qualitative studies, focusing on clinicians’ understanding of the target’s impact suggest that although patient flow and ED experience for staff and patients may have been improved, this has happened at the expense of quality time for communication and treatment [31].

This paper aims to fulfil a gap in the literature by examining changes in clinical and organisational processes that preceded or followed the introduction of an ED wait target. Its main objective is to demonstrate the role of space, time and information technology in the optimisation of patient flows. It does this by examining how these social and technical elements were used to support the 4 hour wait time target in the English NHS and what it means for clinicians to practice emergency care in this environment.

### Study context

The (arbitrary [31]) 4 hour wait target was announced by the English Department of Health in 2000 [32,33], and took effect in January 2005. Without any reference to other equally important sources of ED overcrowding, such as resources, staffing and bed availability [34,35], the idea was that through this target, EDs would be forced to adopt private sector styles of management [36-38] so as to optimise their operations [39], particularly in an NHS of increasing number of ED attendees (from 13.9 m in 1988 to 21.3 m in 2011) and of fewer hospital EDs (from 310 in 1988 to 150 in 2009). Politically, the context for this policy direction was one of the perennial “crises” in the NHS, with extensive media coverage [40] of patients waiting for long periods of time on trolleys in EDs. Consequently, an intensive “reengineering” [41] of emergency care began by emergency clinicians and managers in individual departments [42] who worked to identify bottlenecks, map patient flows (particularly “high volume – low variety” groups of patients) and introduce “fast tracking” care [43,44]. This led to the development of 4 main patient streams (“minor”, “major”, medical and surgical admissions) as a system for reducing waiting [45,46].

This policy-led reconfiguration of time management in ED had to be linked to space and the role of the built environment in supporting patient streaming [47]. To address an evident “lack of fit between layout, activities and staff numbers” [48], two studies, commissioned by NHS Estates [49,50], provided more specific recommendations about efficient ED design layouts. Spatial reconfigurations were undertaken which concentrated on bringing together (or separating) both movement and people, based on whether interactions needed to be minimised or maximised [51,52]. Around the same time, the most ambitious healthcare IT project in the world, the National Programme for Information Technology (NPfIT), began to procure clinical information systems across the NHS [53]. Such systems were intended to ensure collection of accurate data for benchmarking and outcomes improvement [54-56].

## Methods

### Ethical considerations

Our research was approved by the Nottingham NHS Research Ethics Committee (ref. 07/H0408/160). We obtained informed consent from the participants and guaranteed anonymity and confidentiality.

### Design, sampling and data collection

Empirically our findings come from a wider study conducted in the ED at one of the largest hospitals in the UK (146,000 ED attendances per year). In that study, we were interested in identifying factors that contributed to the implementation of an Emergency Department Information System (EDIS). However, it soon became apparent that the 4 hour waiting time target, as well as the spatial redesign of the department (completed around 1.5 years before the official introduction of the target) had created an impetus for acquiring this information system, as a way for the ED to meet their targets and regain control of their expanded physical space.

Following a purposive sampling technique, we conducted our semi-structured interviews over a total period of 8 months (April - November 2008). All the participants were using the system at the time of the interviews and they were working in the department for at least a year before all these changes were completed. Particular questions during interviews focused on (1) how the staff understood their roles in the context of target-oriented emergency care, (2) on identifying the ways it had transformed their practice and (3) their relationships with patients and other colleagues. While observation was not the main data collection method, the project entailed spending a great deal of time in the ED, and a note was made of any interesting and salient data observed.

### Data analysis

Each interview was digitally audio-recorded and all digital audio files were then transcribed, organised and analysed with the use of QSR NVivo 8 software for qualitative data analysis. After the completion of the data coding, the transcripts were reread, contrasted to developed thematic categories and cross-referenced for relevance, consistency and relationships. A final test included a discussion of our findings with the department’s nursing team leader. Both design blueprints (before and after the refurbishment) are discussed to highlight changes necessary for an optimised interaction of time, space, information technology and people under this new model of emergency care.

## Results

The 28 participants in this study (23 female and 5 male) included the system administrator, the change manager, 2 Emergency Department Assistants (EDAs), the operational services coordinator, 4 Emergency Nurse Practitioners (ENPs), 4 charge nurses (NICs) and 15 staff nurses. We analyse the way the introduction of the wait target reconfigured this ED, namely the spatial layout, the flow of patients through the department, the implementation of a new information technology and the flow of power through the clinical and professional relationships of its staff. By highlighting the “high interrelation” [57] of these social and technical aspects, we show how this new arrangement is stabilised, how it redefines and shapes emergency care as well as the unintended consequences of the new time constraint.

### Redesigned Spaces: compartmentalisation

**Figure 1:**
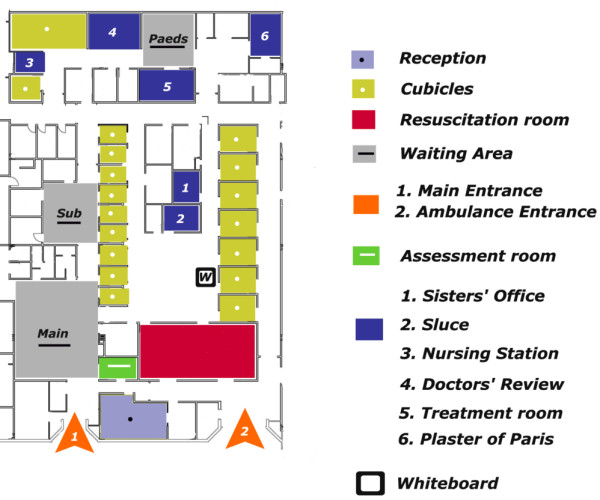
Old layout of ED.

**Figure 2:**
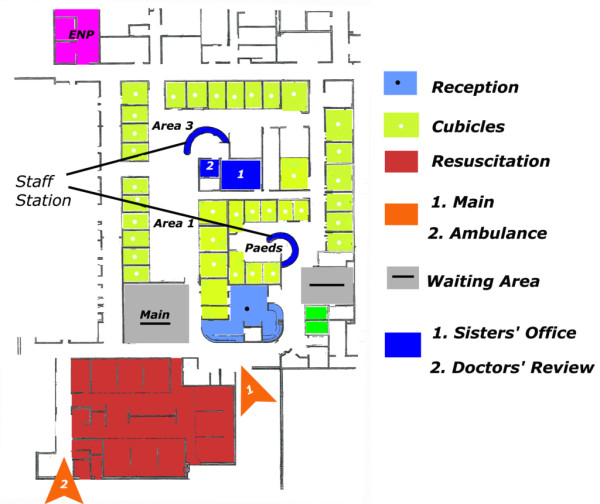
New layout of ED.

The interviewees began by discussing how the physical space of this ED was redesigned. This was because they had been treating an increasing number of ED attendees. There were also issues of security, privacy and dignity for their patients, particularly inside the treatment rooms. They came to the conclusion that the ED building plan and patterns of space usage were good enough for the old service model of treating patients in priority order but not the new “See and Treat” model of patient streaming. They also had to double the number of rooms and, therefore, their capacity to treat patients in dedicated spaces with dedicated staff. However, everything had to be done within the existing physical boundaries of the department. In order to optimise the safe and prompt flow of patients, the department had to be *“compartmentalised”*, meaning that the previous unitary network of ED clinicians had to be broken down into a number of smaller networks of clinical teams and dedicated spaces. Moreover, the new layout had to facilitate better surveillance of all areas and easy way-finding for ED patients and visitors. By fine-tuning all these processes though *integration* or *segregation*, the department was thought to be better equipped to meet performance standards, while creating a satisfactory experience for patients and staff.A closer examination of the design blueprints before and after the change demonstrates this separation of the different areas of the department and how it has since minimised the movement of staff between them. In Figure 1, we see that, previously, clinical areas, especially the central area (for assessing and treating minor and major injuries/illnesses) and the resuscitation room, constituted the main hub where all the activity was taking place. The reception desk and the waiting areas were peripheral to this hub, with the Children’s section at the back of the department. For patients, the reception desk was not directly visible and there was a long public corridor which led them to the two main waiting areas. Another public corridor separated reception from the main area of clinical activity. With these long corridors, natural way-finding was quite difficult for walking patients and visitors. In addition, staff had to walk through public spaces to access other administrative and clinical areas. The U-shape design inside the central area created many problems for movement as well as for maintaining an adequate level of privacy and security for more remote rooms.In contrast, the new layout (Figure 2) necessitated that all clinical areas were placed around the waiting area for patients. Public spaces were integrated and distances minimised so patients walked a straight line to get to treatment areas. Common corridors between the main and ambulance entrances to the resuscitation room were replaced by a more direct and private one. There was also a smaller, private corridor adjacent to Areas 1 (“minors”) and 3 (“majors”) for cases where the ambulance crew needs to transfer a patient directly. EDAs could overlook the entrance and waiting area from the reception desk, as well as control access for patients who enter the two main treatment areas. This arrangement brought the clerical and nursing staff closer together with an internal, private door. Now, all the paperwork could easily be retrieved without distant journeys through public spaces that led to impromptu encounters with patients and, thus, delays in treatment. Similarly, the resuscitation room was brought closer to the ambulance entrance. In this way, nurses could interact with the ambulance crew unobstructed.

This segregation of patients and visitors in one waiting area created more space for Areas 1 and 3. These areas were integrated and connected with the Children’s section via a private corridor, while the old Children’s area was allocated to the new ENPs. The new design layout created more space for accommodating a large number of patients and clinicians. Importantly, it allowed their cooperation in numerous ways with minimum turns of direction and within minimum walking distances. Lastly, the incorporation of a central staff station, where all activities were organised, allowed increased surveillance of patients. From this observatory, everyone was directly visible and reachable, whether it is a patient or a member of staff, facilitating security, safety and monitoring. Consequently, the physical space was more capable of integrating the processes that define an ED for a more unobstructed and prompt patient streaming.

### Reconfigured times: constraints

We previously discussed how emergency care was deemed unsatisfactory because of the long waiting times, particularly for patients with minor injuries or illnesses who were constantly pushed to the back of the queue. The 4 hour wait target was intended to minimise this failure of the system by attempting to control time in emergency care work, often by dividing the overall patient volume into smaller, more homogenised units. However, no matter how well-configured these processes were, the messiness of real-world practice would inevitably interfere. Processes interfered with one another, obstructed the ordered flows of patients and stretched the department’s capability for meeting the target. For example, a patient attending an ED with a presenting complaint could not always be maintained in the same stream for her entire trajectory. Clinicians had to deal with these irregularities on the spot. Therefore, patients were only allocated to streams temporarily. They acted as a first attempt to briefly (and quite vaguely) determine the expected resources and people that would be needed for a particular condition. This was a new managerial task and an *opportunity* for workarounds to best serve patients’ needs.

“I am going to be putting them in the ‘majors’ area and they need to be seen quite quickly, but because they are in ‘minors’ still, or do they automatically become a ‘majors’ because they've got a nasty injury? Or are they still a ‘minors’ because it's an injury? The other one would be with injuries again, you've got your category 6 which is your doctors’ minors and you've got your category 7 which is your ENP and then your category 4 which is your ENP priority, but you've not got a category for doctors’ priority ‘minors’, so they would just go as a 6, if they need to see a doctor and they were an injury, but then how do you put down”; (Clinician 7).

However, the busier it got in the ED, the greater the need to speed up clinical performance. The target, more than actual illness and its urgency, gradually became a critical measure of accountability and, crucially, *the target* had the authority to instigate specific actions.

“Obviously, if there is a patient that needs to stay in the Department because they are unstable or because of their clinical condition, there's a lot of pressure put upon the nurses in charge or the coordinators…that they move them” (Clinician 1).

There were many legitimate reasons why critical (to the target) delays may unfold in the ED, such as waiting for test results or for a specialist to come and see a patient. The ED inevitably required the timely cooperation of many different clinical units and professionals from elsewhere in the hospital. In certain cases, however, processes could be speeded up and those who act on the target’s behalf also got to be its authoritative representatives for ensuring that escalation is completed by all relevant staff members, both within and outside the department. For example, the NIC communicated with the Duty Nurse Manager or with the Bed Manager to move patients, who were close to breaching the target, to the admitting ward or the operating room.

### Bridged spaces and times: information technology

Monitoring and executing emergency care, especially under time constraints, required a new kind of intermediary between space, time and people. The introduction of EDIS came to the rescue of the department which had struggled to figure out how to manage the increasing complexity of their work. It was a technology that can create and maintain, for example, 4 hour wait reports or billing records for tariff-based procedures. It also offered a new, consistent and less confusing way of managing the information derived from keeping track of people, procedures, times and places.

EDIS complemented the restructuring of the department by supporting the new organisational arrangements. In fact, the department had always been keen to have a new system in place because they knew that *“by expanding it physically it was going to become more difficult to manage it practically”*. A much bigger working area, with many more rooms for the increasing number of ED attendees, became easier to handle. This was because EDIS had minimised the *“guess work”* of locating patients amongst different areas of the department, since clinicians could now check the system’s map to see where every patient was.

**Figure 3:**
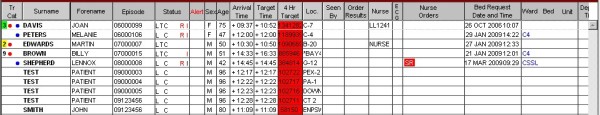
Part of EDIS’ main clinical screen.

Undoubtedly, the system could not be held responsible for meeting the target. This was down to the ED clinicians and how quickly they saw and treated patients. What it did, though, was to increase awareness of time and space for patients and processes by displaying highlighted information on its tracking screen. Patients could now pass through the department in a much more efficient way than previously, *“unless there was somebody specifically on the shift to keep a track of times”* using paper or the metal magnetic strips on the confined space of a whiteboard. It comes as no surprise that the *4 hour target* column (Figure 3), in the table of current attendees, was not only coloured red, amber or green, depending on which patient was about to breach the target, but it was also at the centre of the computer screen, distinctively separating patient and condition-related information (age, sex, triage category and investigations status) from other administrative-related ones (location, assigned clinicians, bed requests).

Therefore, neither EDIS nor clinicians actually ensured the meeting of the target. It was achieved by their collaboration, and how quickly this arrangement of people and technologies adjusted its speed of interactions to the pressure of the volume and acuity of patients. As long as users inputted the necessary data, the system monitored, computed and highlighted the required information. Importantly, the development of this collaboration of people and technology supported the continuous self-management of ED activity. For example, coordinators could check what is happening in other areas and respond quickly to new requirements. Having a *“better picture”* of what was going on in each subsection and in the department as a whole, coordinators were capable of managing staff in real-time and were able to *“redeploy people from different areas to where the biggest workload is”*. This was true, especially after the introduction of the target. EDIS has made the process of managing for the targets possible, *“without it, it would have been a nightmare”*. Although there were, initially, some concerns over issues of *surveillance* of their practice, they now considered the system to be a useful tool for identifying potential bottlenecks that could compromise timely patient care.

“In the early days, it just seems like a big brother tool, they’re monitoring us, … making sure that we’re doing, but then when it actually comes around, and you become one of the people that are managing things, it does enable you, it’s not watching in a bad way. It's a case of it enables you to see it overall what's going on, for tracking patients and seeing where problems lie” (Charge Nurse 5).

New organisational, data-driven, processes, like the weekly *“4 h wait meeting”*, have since been put in place to discuss reasons for target breaches, suggest ways to improve the situation and *“alleviate the pressure”*. After accepting their designated managerial role, clinicians were now locked into it. Gradually, they started internalising the values and outcomes of accountability, characterised by the production of more accurate information.

### Shifting tasks

We previously described how the target had developed into an impetus for change in EDs by restructuring and reordering the organisation, based not necessarily on professional groups, but on the departments these clinicians belong to. All staff members had now been enrolled and mobilised to pass pressure on, both inside and outside the department according to *“a very strict plan of action for patients who are nearing or will breach”* the target. It is this escalation of accountability, by skilful coordinators, that became the motivating force for action. Putting aside long-standing professional hierarchies, nurse coordinators, for example, could now *“ring and harass”*, *“shout down”* or *“badger”* (speciality) doctors until they fulfilled their role in this process for the next operational step to take place. Otherwise, their unavailability would be put down as the reason for the target breach.

“ [The target] gives you a bit of ammunition really because at first, everyone just thought, oh, it’s an ED target, we don't need to worry about it. When actually it's a hospital target and they do need to worry about it and once they realise this, they’re actually getting more helpful” (Clinician 4).

Inevitably, the work of each professional group has changed as the system in which they work changed. Consequently, any conflicts over the development of new healthcare roles moved from the ‘ideological’, to consideration of measurable outcomes, which now provided the basis for decisions. In EDs, the new professional role of the ENP, a specialised nurse for the purpose of taking up mundane tasks and releasing time for doctors, was developed to strengthen the focus on the target. These nurses were trained to act autonomously, based on protocols, in health promotion, education, assessment, diagnosis and interpretation of X-Rays, while they can treat and prescribe medications for minor illnesses and injuries [58,59]. They are now considered an effective solution for reducing wait times, particularly in overcrowded urban EDs with high volumes of low acuity patients and physician shortages [60]. Most of the interviewees in our study thought ENPs made an invaluable contribution to the reduction of target breaches.

We have already seen how the focus on the target as a means of addressing the chronic problem of ED wait times led to the replacement of one big queue, in which every patient was prioritised, with a smaller, more manageable, and less visible queue. In conjunction with the new system, an added benefit of this change was that these patients could have more information regarding their position in this queue which *“does help them”*. For example, patients waiting could be informed about how many people were in front of them. EDAs at the reception, while they could not know precisely how long a patient would have to wait, could look up the queue in EDIS and reassure these patients that they were *“still on the system, everything is in time order”* and that they would not *“get missed”*. On the other hand, this was only for those patients who are accepted into these queues. Just like the clinicians who managed their trajectories, patients were subjects of the same target. The target acted as the objective justification for exclusions. Patients, whose medical condition did not meet the profile of the ED attendee, were referred to other services (e.g. GPs, minor injury units and walk-in centres).

“Before, we couldn't have sent anybody away, we didn't have that sort of authority to send people away, so it was like well…you're not important to be seen, so everyone needs to be seen before you, so if you're waiting here 6 hours that's how long you will wait” (Clinician 5).

For those patients who had successfully managed to navigate themselves through the maze of the healthcare system and had been given a ‘boarding pass’ to the ED, a better clinical experience and quality of care was “pledged” [61]. This was evident from the fact that almost all of our participants stated that they would not want to go back to the previous clinical reality of EDs with “*doctors sitting on the floor doing assessments”* and patients *“who had been waiting two days to get to a ward”*. On the other hand, this more modern, target-oriented, healthcare inevitably happened at the expense of some of the clinicians’ more traditional values around their personal relationships with the patients and their colleagues. They were forced to redefine and restructure their system of values to be more managerial. More speed, more compartmentalisation and more technologically-mediated communication sometimes made them “*feel under pressure to quicken up”*, which in turn made it *“very hard not to sacrifice patient care”*.

“People don’t speak to each other about patients much anymore. [EDIS] takes you away from the patient. We used to…go in with the doctor to find out what was going on with the patient and then the doctor would relay it to you, so you’d learn from the doctor… now it’s all just on screen and people don’t talk about patients…it’s all just conveyor belt, it feels more conveyor belt and that’s obviously what they wanted with the efficiency and the four hours and every nurse will tell you that”. (ENP 5)

## Discussion

We did a qualitative study with the aim of understanding the transformation of clinical practice and local consequences from the introduction of a national target for waiting at an ED. We found the 4 hour wait target supported the development of a new type of spatial and temporal regulation of ED staff’s work. This was achieved by first redesigning the built environment into separate areas, according to acuity categories, so as to facilitate efficient patient throughput. ED staff perceived this compartmentalisation as an improvement for security, privacy and way-finding for patients, and it reduced unnecessary movements. Moreover, they considered the target a catalyst for building collaboration with the rest of the hospital and for speeding up clinical performance. Irrespective of professional hierarchies, they were more likely to be heard when they requested a specialist opinion and inter-departmental efforts were made to secure beds for admissions promptly. The target has also increased the value of the ENP’s role by autonomously treating patients with minor illnesses/injuries. It also necessitated the implementation of a new clinical information system. ED staff found the system’s capabilities for advanced tracking, awareness of potential target breaches, and continuous, real-time management of staff particularly useful. These features were also helpful in reassuring the patients that they would be treated in time. The convenience of accessing structured information and of producing reports led to the development of new self-regulating processes, such as the 4 hour wait target weekly meeting. However, some nurses felt that the added pressure to move patients quickly has affected their relationships with patients and colleagues.

We have been able to make explicit the social and technical aspects of emergency care and highlight the complexity of their interrelations. Thus, we were able to tell a more complete story of the process by which the new policy of the 4 hour wait target and an emergency department adapted to each other at the local organisational and professional context [62,63]. We paid particular attention to unintended consequences as they revealed the strength of mutual dependencies between the social and technical elements that hold this new way of working together [64] and, provided an opportunity to investigate their role in shaping the outcomes of this organisational change [65]. In this case, it helped us to understand how space, time and information technology can be manipulated and mobilised. From there, we described a process by which they shape and are shaped, locally, as the new arrangements struggle to reach a consensus around the wait target and the ED consolidates itself as a ‘modern’ emergency department.

Reducing maximum waiting times in ED has been the focus for this policy, as they are known to be important to patients, and are easily measurable, understandable (unlike, for instance, quality and safety) and easier to achieve (unlike average waiting times) [66]. On the other hand, the ED has traditionally been a resource-poor and comparatively neglected area of the hospital, despite its high public profile. This is partly due to the low status of ED work within the wider medical profession [67], and a perception that, despite the major emergencies, much of the ED’s work consists of minor injuries and illnesses. The target meant that the ED, often for the first time, became the focus of managerial attention and resources [68]. The system of performance management in the NHS meant that hospital Chief Executives and Boards were directly accountable for the performance of the ED against the target, and therefore took a much closer interest in the ED than had been the case hitherto. There was a concomitant expansion in the resources available to EDs. For instance, though the redesign had happened prior to the introduction of the 4 hour wait target in 2005, its announcement in 2000 and the subsequent work of emergency departments on fast track care made the reconfiguration of space a necessity. Likewise, the introduction of the IS system, and the streaming processes were all originally introduced in order to meet the target, but collectively led to a revolution in working in the ED.

In particular, the redesign of the built environment, towards compartmentalisation, signifies an important paradigm shift on the way healthcare organisations understand the practical value of space in the mediation of work. They acknowledged, perhaps for the first time, that spaces are not just neutral containers of social action. Therefore, if the aim is to implement a certain model of healthcare delivery, the configuration of the physical environment becomes a precondition, as “function follows form”. Likewise, time is not fixed and absolute. It too exerts meaning and it is embedded in local contexts and processes, structuring actions, events and behaviours. But when it comes to organisational productivity, the quantifiable clock time is mostly viewed as a simple, independent, self-explanatory variable and a resource that can be manipulated accordingly so as to increase efficiency of work.

Here, the introduction of the 4 hour wait target brought a new reckoning and re-embedding of time in the ED. Under this new “temporal rhythm”, patients arrived with a well-defined “temporal trajectory” of their condition while staff had a very “close and inflexible time horizon” to complete activities [69]. Any delay could cause the ED to (unjustifiably) exceed the 2% exceptions margin on target breaches. As ownership of the target moved across the hospital [14] more measures were taken to improve flows and minimise bottlenecks. Since the target was introduced, there has been, for instance, substantial growth in the number of emergency medicine consultants, development of new clinical specialities for treating minor injuries (ENP) [24,43] as well as increased leadership, particularly for nurses, who now have an enhanced role in care coordination. In effect, the target brought about a change in the ED’s relationship with the rest of the hospital. There was a major shift in the balance of power [70] between the ED and other hospital departments. We offer striking evidence of ED staff *arguing up* the hospital hierarchy and pushing for specific actions to take place so as to speed up care [71] and prevent a target breach. Pressure on nurses to meet targets was passed onto those they consider (partly) responsible for the breaches (doctors in inpatient specialities) [14].

Moreover, our findings demonstrate how the new technology of EDIS came to support an increasing need for the ED to accumulate and remotely display more information so as to track patients and coordinate activities [72]. Through a more efficient “horizontal” and “vertical” surveillance [73], it has become an essential aspect of the new model of target-oriented clinical teamwork. Importantly, it has also contributed to the reconfiguration of inter-professional power relationships. By taking up the sequencing activities, EDIS acted as a reliable and independent ‘observer’ who provided the shared temporal order necessary for work synchronisation. In effect, it equalised power relationships with fewer work-related conflicts between these two groups [74]. This is because the meanings and purposes of organisational activities, and boundaries are redrawn as everyone gets synchronised to the technology’s temporal rhythm [75].

While the new resources and shift in the balance of power in ED’s favour were viewed positively by the ED staff, other unintended consequences of the target were more unwelcome. We did not find evidence pointing to any change of the type or quality of care [21], but clinicians were concerned about how the target had affected their ways of working. They felt like they had less time with their patients, and were under more pressure to keep moving them through the department [27,31,76]. Here, our qualitative findings complement those of other studies [77-80], which have statistically confirmed an increase in clinical activity around 20 minutes before the 4 hour cut-off since the introduction of the target. Undoubtedly, clinicians do not wish to go back to the era when patients waited for many hours before they were treated [31]. However, they viewed their work as becoming more like working on a production line (indeed, that metaphor appears in several of the interviews), as they gradually adopted a “distal” healthcare paradigm of technically managing the business side of their practices [81]. This could be a manifestation of “proletarianization” [82]. This is the ‘modern’ process by which organisations seek to transform the work of professionals, who typically have a high degree of independence and discretion, into work where they are much more closely monitored and supervised, aligning their work practices much more closely with the organisation’s requirements.

In this case, the *modernisation* of EDs began by translating patient dissatisfaction with wait times into an “internal” performance indicator [83]. It signified the “pressure of time” [39] as a decisive characteristic of healthcare efficiency and a hard to refute “political symbolism” [83]. Consequently, this new “professional ethos of self-governance” [84] required the internalisation of the values of responsibility and accountability [85]. The more ED clinicians internalised them, the more their capacity for self-governance and learning increased. However, to achieve this, the ED has been arranged and steered towards the production of more information as a way of meaningfully interpreting the target and optimising its processes so as to improve emergency care. These include better bed management systems, protocols and guidelines for speeding up treatments, the extension of nursing responsibilities for undertaking more biomedical, managerial and administrative activities, the application of time limits for specialty doctors to attend ED from other parts of the hospital [86], the technological mediation of communication [87], and workload management systems [88]. Such efforts at standardising care, which involve processes, information systems and the physical space, have intensified lately as more EDs embark on *Lean* process improvement methods. While these initiatives may hold a great potential for addressing lengths of stay and patient satisfaction, the added, “indirect” [89] burden they placed on clinicians in terms of workload, autonomy and anxiety is often neglected. Thus, while the new way of working was successfully and sustainably stabilised (and continues to the time of writing), this stabilisation was not without wider social consequences for the ED and the staff within it. Individual clinicians continue to experience a stark conflict between the two ethos (traditional clinical and new professional) in the process of improving the quality of care.

### Limitations

The study was limited to one emergency department. Generalisations should thus be made with caution. The sample was of appropriate size given the nature of the topic and, in particular, difficulty in recruiting participants due to high levels of workload and staff turnover. The decision to recruit mainly nurses was based on the fact that this professional group represented the biggest user group of this system, which is also responsible for the coordination of activities in this clinical setting to meet the wait target. Also, our attempts to recruit medical staff that met our selection criteria were unsuccessful. We acknowledge that this may be a significant weakness of our sampling methodology.

## Conclusions

Policy changes can have deep and unintended consequences for health care organisations. We have shown that the imposition of a wait-time target led to the development of a new, and very sophisticated, way of working in the ED studied. This consisted of a complex arrangement of people, process, technology and space, none of which was intended by those who originally framed the 4 hour wait target. There is wide agreement among clinicians that this target raised the profile of the ED in the hospital and concentrated efforts to address patients’ dissatisfaction with waiting times. It forced them to self-examine their practices, and rethink about the way they use space and manage information and patient flows. At the same time, it has put added pressure on them which causes concern over the effect it might have on their interpersonal relationships with their patients and colleagues. Linking patient satisfaction with clinician satisfaction may be the way forward.

## Competing interests

The authors declare that they have no competing interests.

## Authors’ contributions

PV designed the study, collected, analysed and interpreted the data, and drafted the manuscript. ST conceived the study, participated in its design and coordination, and helped to draft the manuscript. All authors read and approved the final manuscript.

## Pre-publication history

The pre-publication history for this paper can be accessed here:

http://www.biomedcentral.com/1471-227X/14/12/prepub
